# Image-to-Image Translation for Simplified MRI Muscle Segmentation

**DOI:** 10.3389/fradi.2021.664444

**Published:** 2021-07-06

**Authors:** Michael Gadermayr, Lotte Heckmann, Kexin Li, Friederike Bähr, Madlaine Müller, Daniel Truhn, Dorit Merhof, Burkhard Gess

**Affiliations:** ^1^Department of Information Technology and Systems Management, Salzburg University of Applied Sciences, Salzburg, Austria; ^2^Institute of Imaging & Computer Vision, RWTH Aachen University, Aachen, Germany; ^3^Department of Neurology, RWTH Aachen, University Hospital Aachen, Aachen, Germany; ^4^Department of Neurology, Inselspital Bern, Bern, Switzerland; ^5^Department of Diagnostic and Interventional Radiology, University Hospital Aachen, Aachen, Germany; ^6^Fraunhofer Institute for Digital Medicine MEVIS, Bremen, Germany; ^7^Department of Neurology, Evangelisches Klinikum Bethel, Universitätsklinikum OWL, Bielefeld, Germany

**Keywords:** MRI, muscle, fatty-infiltration, thigh, generative adversarial networks, convolutional neural networks, segmentation, image processing

## Abstract

Deep neural networks recently showed high performance and gained popularity in the field of radiology. However, the fact that large amounts of labeled data are required for training these architectures inhibits practical applications. We take advantage of an unpaired image-to-image translation approach in combination with a novel domain specific loss formulation to create an “easier-to-segment” intermediate image representation without requiring any label data. The requirement here is that the task can be translated from a hard to a related but simplified task for which unlabeled data are available. In the experimental evaluation, we investigate fully automated approaches for segmentation of pathological muscle tissue in T1-weighted magnetic resonance (MR) images of human thighs. The results show clearly improved performance in case of supervised segmentation techniques. Even more impressively, we obtain similar results with a basic completely unsupervised segmentation approach.

## 1. Introduction

Within the last few years, deep neural networks showed impressive performance and gained popularity in the field of radiology. However, the requirement for large amounts of labeled data for artificial neural network training still inhibits practical applications. Since three-dimensional (3D) data requires complex models, this is particularly challenging in radiology. In addition, voxel-based 3D data annotation is highly time consuming. Another challenging aspect is given by an often high variability within radiological data. Although variability due to the imaging setting can be compensated by methods such as bias field correction (1) and contrast adjustment (2), semantic variability caused by pathological modifications is hard to compensate.

Due to emerging techniques, such as fully convolutional neural networks (3) and adversarial networks (4), image-to-image translation has recently gained popularity (5–7). These methods enable, for example, a translation from one imaging modality to another (such as MRI to CT and vice versa) (8). Conventional approaches require image pairs (e.g., pairs consisting of a CT and an MRI scan of the same subject) for training the translation models (5, 6). To overcome the restriction of training based on image pairs, unpaired approaches were introduced (7, 9, 10) and also applied to radiology (8, 11, 12). These models only require two data sets, one for each of the modalities [e.g., computed tomography (CT) and magnetic resonance imaging (MRI)]. As image pairs are often not achievable or at least very difficult and expensive to collect, this opens up completely new perspectives for many radiological application scenarios. For example, if trained models (and especially manually annotated training data) are available for one modality only, data collected based on a different imaging setting can be translated to this modality and can be subsequently processed without further annotation effort.

In this paper, we do not consider a translation from one imaging modality to another using cycle-GAN (7). Instead, we consider a scenario where a certain domain (i.e., a subset of the available data; e.g., non-pathological data) is easier to segment than another domain (13). Image-to-image translation can be applied here to translate from a hard-to-segment image domain to an easy-to-segment domain. If translation is performed appropriately, this approach has the potential to facilitate further processing (here segmentation) and thereby enhance accuracy (e.g., segmentation accuracy) to reduce the amount of required annotated training data or even to facilitate fully unsupervised segmentation.

### 1.1. Thigh Muscle Segmentation

**Figure 1:**
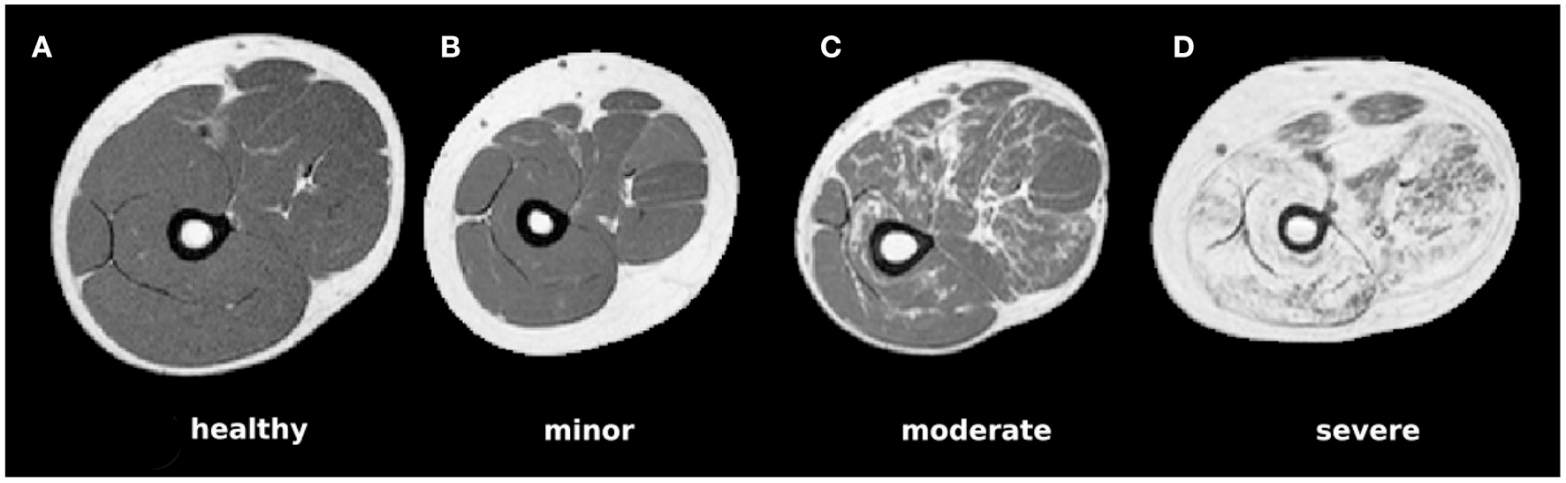
Example MRI slices for each of the four considered pathological categories showing **(A)** healthy muscle only, **(B)** pathological muscle without visible fatty infiltrations, **(C)** moderate infiltrations, and **(D)** largely affected muscle areas.

Muscular dystrophy is a class of diseases caused by inherited mutations in genes encoding for proteins that are essential to the health and function of muscles. They are characterized by a degeneration of muscle tissue, which in muscle imaging appears as so-called fatty infiltration (see Figures 1C,D for example MR images). A relevant disease marker is especially given by the so-called fat fraction capturing the ratio between fatty-infiltration and original muscle tissue volume. For computation of the fat fraction, it is crucial to segment the overall muscle tissue including fatty infiltrations. Although a segmentation of healthy muscle tissue (see Figure 1) can be obtained easily based on thresholding, difficulties arise in case of severely fat-infiltrated muscle as fatty degenerated muscle tissue cannot be distinguished from subcutaneous fat based on the image's gray values (14) (Figure 1D). This problem has been recently addressed in a few studies. Origiu et al. (15) developed an active contours model to detect the muscle boundary and a fuzzy c-means method to distinguish muscle from fat. Gadermayr et al. (14) combined graph-cuts and level-set approaches with statistical shape models. Yao et al. (18) made use of two neural networks to first detect the fascia lata and also incorporate region-based information to finally utilize an active contours method. Although showing best segmentation performance, the latter approach as well as further ones (16–18) are optimized and evaluated on an easier scenario, because all tissue inside the fascia lata is labeled as muscle (apart from the bone).

### 1.2. Contributions

In this work, we make use of a new procedure for facilitating segmentation tasks in order to boost segmentation accuracy. In our approach, a hard segmentation task is mapped to an easier (intermediate) segmentation task by means of unpaired image-to-image translation making use of a cyclic GAN (7). We consider the segmentation of MR images of human thighs showing fatty infiltrations, which are translated to easy-to-segment non-pathological images. For segmentation, we consider methodologies that proved to be effective in previous works (14, 15, 18). Even though we were unable to investigate each individual configuration, we focus on covering a broad range of techniques, namely a pixel-based unsupervised approach, a region based method, a region-based method using shape prior, and a convolutional neural network.

## 2. Materials and Methods

In this work, we first perform image-to-image translation to convert a hard-to-segment into an easy-to-segment domain (section 2.1). After conversion to the intermediate “easy” representation, only the generated fake image is segmented (section 2.2) and the obtained mask is simply mapped to the original image without making any changes.

### 2.1. Image-to-Image Translation

Supposed we have a set of images ${\left\{h_{i}\right\}}_{j=1}^{N}$ of a “hard” domain (H), which are difficult to segment, as well as a set of images ${\left\{e_{i}\right\}}_{j=1}^{M}$ of an “easy” domain (E). Although the underlying distributions (based on the empirical ones *e* ~ *p*_*data*_(*e*) and *h* ~ *p*_*data*_(*h*)) are different, we assume that the underlying distribution of the corresponding ground-truth segmentations *s* (*s*_*e*_ ~ *p*_*data*_(*s*(*e*)) and *s*_*h*_ ~ *p*_*data*_(*s*(*h*))) is similar. Then it follows that, based on a segmentation only, the domain of an image (H vs. E) cannot be predicted with a higher accuracy than chance. Thus, the translated images could also become indistinguishable even if the segmentation mask stays the same, which is the crucial criterion for this approach. Otherwise, in a GAN setting, the generator would be forced by the discriminator to change the object's shape with the implication that the segmentation of the original H domain image would not be the same as for the fake E domain image. As we finally directly map the obtained segmentation mask from the fake E to the real H domain image without making any changes, the similarity of the object's shapes is a strong requirement. Inspecting the considered MRI data, we notice high variability between patients in general but no systematic differences in the shapes between the datasets.

**Figure 2:**
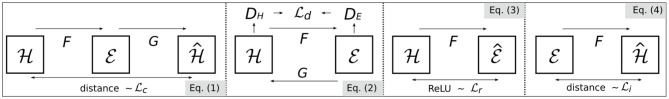
An illustration depicting the individual losses $L_{d},L_{c},L_{i},L_{r}$ contributing to the overall loss on a high level perspective.

Now we focus on a domain adaptation from H to E by performing image-to-image translation, specifically by means of a cyclic GAN (7). This method requires only one dataset for each domain without corresponding pairs. During GAN training, two mapping functions, F:H→E and G:E→H are trained optimizing a combination of a cycle consistency loss


(1) $$\begin{array}{r} \begin{array}{r} L_{c}={𝔼}_{e\sim p_{data}\left(e\right)}\left[{\left\|F\left(G\left(e\right)\right)-e\right\|}_{1}\right]+\\ {𝔼}_{h\sim p_{data}\left(h\right)}\left[{\left\|G\left(F\left(h\right)\right)-h\right\|}_{1}\right] \end{array} \end{array}$$

as well as a discriminator loss


(2) $$\begin{array}{r} \begin{array}{r} L_{d}={𝔼}_{h\sim p_{data}\left(h\right)}\left[\log\left(D_{H}\left(h\right)\right)+\log\left(1-D_{E}\left(F\left(h\right)\right)\right)\right]+\\ {𝔼}_{e\sim p_{data}\left(e\right)}\left[\log\left(1-D_{H}\left(G\left(e\right)\right)\right)+\log\left(D_{E}\left(e\right)\right)\right] \end{array} \end{array}$$

encouraging indistinguishable outputs (based on the discriminators *D*_*H*_ and *D*_*E*_). As the underlying distributions of ground-truth segmentations *s*_*h*_ and *e*_*e*_ are similar, and as there is a correlation between image information and the ground-truth segmentation (which is a natural requirement for all segmentation applications), it can be expected that during image-to-image translation using a cyclic GAN (7), the images are translated from domain H to E without changing the semantic structure in the image (i.e., the shape of the muscle). To account for the specific application scenario, we introduce a further loss function based on the rectified linear unit (ReLU) *r*


(3) $$\begin{array}{r} \begin{array}{r} L_{r}={𝔼}_{h\sim p_{data}\left(h\right)}\left[r\left(F\left(h\right)-h\right)\right]+\\ {𝔼}_{e\sim p_{data}\left(e\right)}\left[r\left(e-G\left(e\right)\right)\right], \end{array} \end{array}$$

where *r*(*x*) = *max*(0, *x*). This method is introduced in order to account for the fact that healthy muscle tissue in MR images shows a lower voxel value than pathological muscle tissue. For this purpose, if muscle tissue is translated from H to E, voxel values should not increase, but only decrease. Vice versa, from E to H, voxel values should only increase and not decrease. By adding this further constraint, we expect that the overall structure and consequently also the segmentation could be maintained more effectively. This domain specific loss is finally combined with the identity loss


(4) $$\begin{array}{r} \begin{array}{r} L_{i}={𝔼}_{e\sim p_{data}\left(e\right)}\left[{\left\|F\left(e\right)-e\right\|}_{1}\right]+\\ {𝔼}_{h\sim p_{data}\left(h\right)}\left[{\left\|G\left(h\right)-h\right\|}_{1}\right] \end{array} \end{array}$$

to focus on maintaining the morphology and to ensure that data from the easy domain E does not get extremely dark due to $L_{r}$. All utilized losses are summarized in Figure 2.

### 2.2. Segmentation

For segmentation, we make use of four methods that were applied to muscle segmentation tasks. Due to the rather small amount of data for training, we focus on the following methods that can be effectively trained with a small amount of data. The first approach is based on the **Gaussian Mixture Model (GMM)**, which is fitted to the data in order to identify clusters of three different classes: muscle, fat, and bone/vessels. Initial cluster centers are fixed to the minimum gray value (*s*_min_), maximum gray value (*s*_max_), and finally a value in between ($s_{\text{min}}+\frac{s_{\text{max}}-s_{\text{min}}}{6}$). This method is completely unsupervised and does not require any training data. In order to incorporate boundary smoothness constraints, we furthermore investigate a probabilistic **Graph-Cut (GC)** technique (the initialization is obtained by the GMM and the probabilistic model is trained based on ground-truth annotations). To additionally incorporate a statistical shape model, we make use of the **Shape-Prior Graph-Cut (SPGC)** approach (14). In this case, the shape model (which is optimized for small data sets) is trained by estimating a probability map for each pixel after an initial registration (leading to excellent performance for pathological images). SPGC and GC both require annotated training data as the probabilistic model need to be trained on ground-truth data. Details on these approaches are provided in (14). As reference for a state-of-the-art **convolutional neural network (CNN)** approach, we apply a 2D U-Net (3) including a GAN-Loss, also referred to as Pix2Pix network (5). In this data-driven approach, a segmentation model (implicitly including a shape prior) is automatically learned during optimization of the weights of the convolutional neural networks.

### 2.3. Experimental Details

The T1-weighted MR images were acquired on a 1.5 Tesla Phillips device with fixed echo time (17 ms), bandwidth (64 kHz) and echo train length (6) and a relaxation time between 721 and 901 ms. The sampling interval was fixed to 1 mm in x-y-direction and 7 mm in z-direction. Bias-field correction was applied to compensate homogeneity (19). Similar to (14, 18), the data are separated into the four categories “healthy,” “minor,” “moderate,” and “severe” corresponding to the degree of fatty infiltration. As the categories “healthy” and “easy” can be rather easily segmented with existing approaches (14), they are not considered during evaluation. Healthy (and easy) scans could also be translated with the proposed pipeline, but remain almost unchanged. Binary ground-truth was acquired to cover muscle volume only, also excluding small fascias (**Figure 4a**). Due to high correlation of consecutive slices and to limit manual effort, each forth slice (transversal plane) was annotated under strong supervision of a medical expert (Madlaine Müller). For parameter optimization of the segmentation stage, grid search combined with leave-one-out cross-validation is applied to determine the best combination individually for both datasets. The parameters of the graph-cut approaches consist of curvature weight λ_s_ ∈ [0.001, 0.002, 0.05, 0.1, 0.2, 0.5], low-pass filtering weight σ ∈ [1, 2], shape prior weight λ_sp_ ∈ [0.1, 0.2, 0.5, 0.7, 1], and neutral probability *p*_n_ ∈ [0.2, 0.3, 0.4, 0.5]. The CNN segmentation approach is trained for 200 epochs with learning rate 0.0002 for each setting and each fold. Fourfold cross-validation is conducted. For data augmentation, random cropping (256 × 256 patches from images padded to 300 × 300 pixels), rotations with multiples of 90° and flipping is applied. For further parameters, we use the defaults from the pytorch reference implementation.

**Figure 3:**
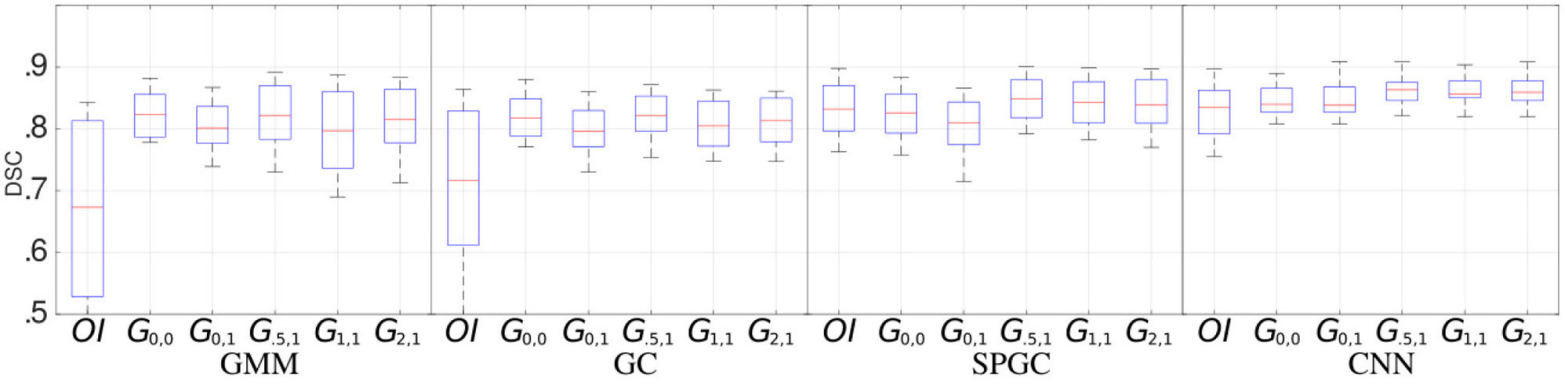
Segmentation performance (median, quartiles, min, and max DSCs) for the four segmentation approaches (GMM, GC, SPGC, CNN) and for individual GAN configurations (*G*_*n,m*_) compared to a direct segmentation (i.e., segmentation without image translation) of the original image data (*OI*). The indices of GAN-based methods define the loss weights *w*_*r*_ (first index) and *w*_*i*_ (second index).

For image translation, a cyclic GAN (based on a ResNet with 9 blocks as generator and the proposed patchwise CNN as discriminator) (7) is trained for 200 epochs with learning rate 0.0002 based on a “hard” and an “easy” dataset. The “easy” dataset contains 2D slices showing “healthy” and “minor” data both showing no visible fatty-infiltrations and the “hard” dataset contains “moderate” and “severe” images. The individual sets are merged to maximize the number of training images (overall, we obtain 649 “hard” (from 19 patients) and 1,124 “easy” 2D images (20 patients) with a size of 256 × 256 pixels). The losses $L_{d}$ and $L_{c}$ are equally weighted (*w*_*d*_ = 1, *w*_*c*_ = 1) (7). For *w*_*i*_ and *w*_*r*_ (corresponding to $L_{i}$ and $L_{r}$), several relevant parameters are evaluated as shown in Figure 3. The standard GAN setting is evaluated with *w*_*i*_ = 0 and *w*_*i*_ = 1 (*G*_0,0_, *G*_0,1_) and three settings for *w*_*r*_ > 0 are evaluated with *w*_*i*_ = 1 (*G*_.5,1_, *G*_1,1_, *G*_2,1_). In the latter case, the identity loss is required in order to prevent the GAN from generating extremely dark fake-“healthy” MRI scans.

## 3. Results

Figure 3 shows the segmentation performance individually for the four segmentation methods (GMM, GC, SPGC, CNN) and for the different GAN configurations (*G*_*n,m*_, with *n* and *m* defining the weights such that *w*_*r*_ = *n* and *w*_*i*_ = *m*). For completely unsupervised segmentation using GMM, the baseline relying on original images (*OI*) is outperformed clearly. The best median DSCs are obtained with the GAN setting *G*_0.5,1_ (DSC: 0.82 compared to 0.67 in case of *OI*). A similar effect is observed for GC. The benefit of image translation is clearly smaller in case of SPGC and CNN. For all configurations, *G*_0.5,1_ exhibits the best DSCs with scores of 0.85/0.82/0.86 compared to 0.83/0.72/0.83 in case of *OI* and 0.83/0.82/0.86 in case of the standard cycle-GAN configuration *G*_0,0_ for the approaches SPGC/GC/CNN.

**Figure 4:**
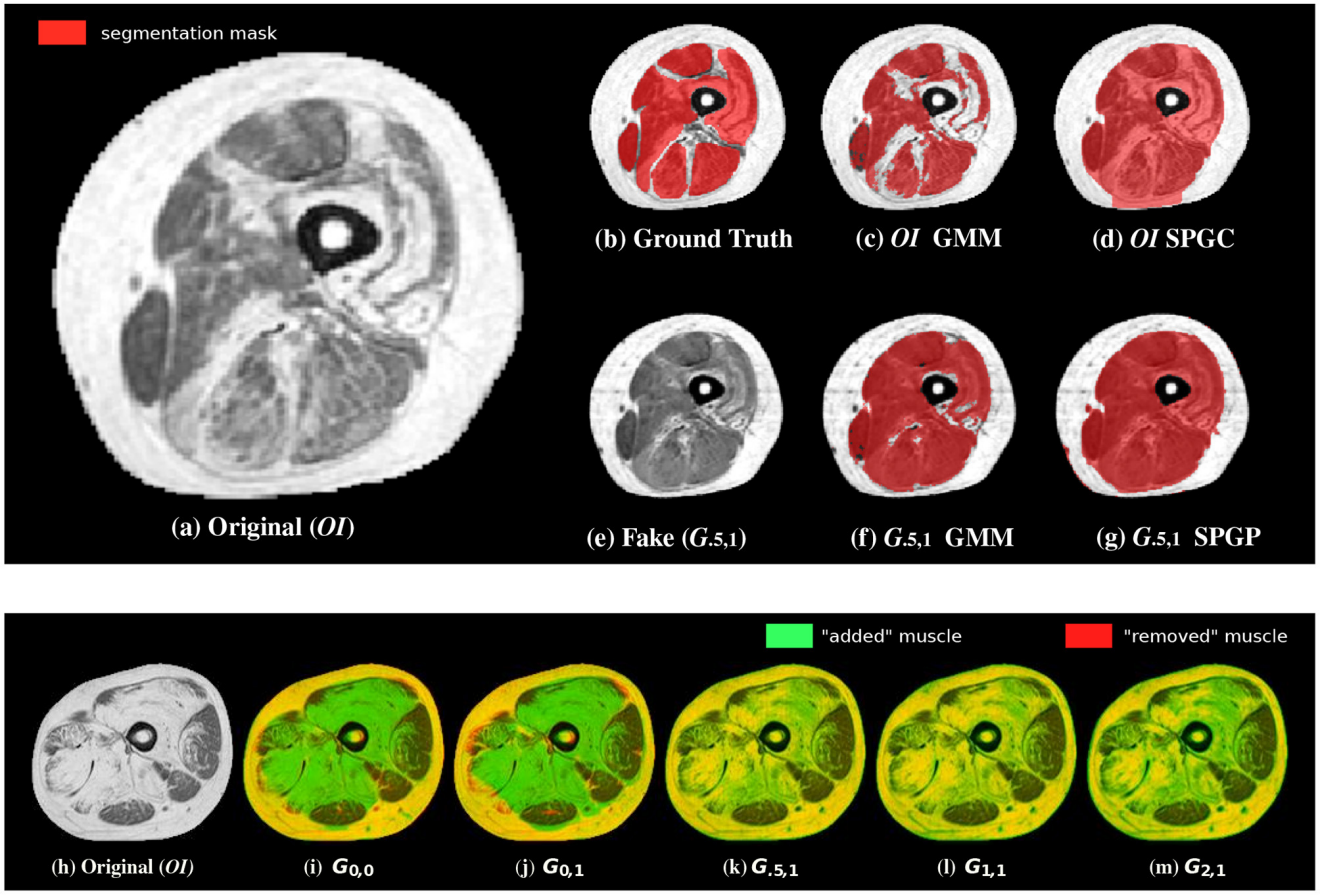
Example segmentations **(c,d)** of the original image **(a)** as well as of the translated images **(e–g)** in comparison to the ground-truth annotations **(b)**. Although small structures often cannot be completely reconstructed (especially SPGC leads to over-smoothed masks), overall segmentation robustness increases in case of the translated image **(f,g)**. The bottom row shows an overlay of an example original image **(h)** with the corresponding translated images. Although green color indicates “added” muscle tissue, red color indicates “removed” muscle. Yellow shows unchanged intensities. The configurations without $L_{r}$ show removed muscle tissue and also added muscle in wrong areas **(i,j)**. This is not the case when including the novel domain specific loss **(k–m)**.

Example image translation output and example segmentations for GMM and SPGC are provided in Figures 4a–g. Results show clear improvements for the rather basic methods GMM and GC, which fail in case of original pathological data. For the methods that are capable of learning the shape of the muscles, even with *OI* median scores above 0.84 are achieved. Even for SPGC and CNN further improvements are achieved in case of image translation (CNN: 0.86 compared to 0.83). The bottom row of Figure 4 additionally shows the impact of different image translation settings for an example image.

## 4. Discussion

Making use of unpaired image-to-image translation, we propose a methodology to facilitate segmentation tasks for specific scenarios where a hard problem can be mapped to an easier task. The most impressive performance gain is observed in case of fully unsupervised segmentation (GMM) applied to the “severe” data, which was expected due to the high degree of fatty infiltrations complicating a pixel-level classification without contextual knowledge. However, also with probabilistic graph-cuts with (GC) or without a statistical shape model (SPGC) and even for the deep learning based approach (CNN), a slight increase of performance with image translation is observed. For the latter, this is not completely obvious since the segmentation network should be capable of learning the same invariance to pathological data as the translation model. However, for learning the translation model, all available data could be used and not only the annotated data (each forth slice only), which is supposed to be a clear advantage due to the small training data sets. Related work investigating a similar application in digital pathology also suggests that two individual networks performing a task in two steps can be advantageous (20).

Considering the different GAN configuration, we note that especially the introduction of the new loss $L_{r}$ leads to best median DSCs and the configuration *G*_.5,1_ is never outperformed by any other GAN configuration.

By considering the qualitative results (Figure 4), we note that the converted images (in case of *G*_.5,1_) actually exhibit a high similarity compared to data of healthy subjects and most importantly they finally lead to improved segmentations. Only in some severe cases, it can be observed that the muscle's shape is slightly changed and that small structures are not reconstructed perfectly eventually also affecting the overall segmentation performance. Therefore, we expect that increasing the amount of unlabeled training data can help to improve the image-translation process in order to boost the overall performance of (unsupervised) segmentation even further.

For clinical application, we estimate that a DSC of between 0.85 and 0.90 is required for reliable diagnosis. Visual inspection can help to quickly identify scans for which segmentation failed. After image translation, rates below 0.85 only occurred for severely affected patients.

To conclude, we proposed a methodology to simplify segmentation tasks and thereby boost the segmentation accuracy by mapping a hard segmentation problem to an easier task. For means of enhancing the image-to-image translation approach, we introduced a further domain specific loss function included in GAN training. We considered an application scenario on segmenting MRI scans of human thighs and showed that the proposed approach can be effectively applied to either increase the segmentation performance of supervised segmentation techniques, or even to obtain highly reasonable outcomes with completely unsupervised techniques. We assess the latter case as even more relevant with most significant boosts in DSC (up to 0.15). We are confident that this approach is not limited to the considered application but can be effectively applied to other tasks in radiology as well.

## Data Availability Statement

The data analyzed in this study is subject to the following licenses/restrictions: We are planning to make the data set publicly available either upon request or via a publicly available link. Requests to access these datasets should be directed to michael.gadermayr@fh-salzburg.ac.at.

## Ethics Statement

The studies involving human participants were reviewed and approved by University Hospitel RWTH Aachen. The patients/participants provided their written informed consent to participate in this study.

## Author Contributions

MG and BG primarily designed the study. DM, MM, FB, and DT provided valuable feedback and suggestions for improvements from technical and medical perspective, respectively. KL, LH, and MG were involved in technical implementations. BG, MM, and FB were involved as medical advisors. DM was involved as technical advisor (image analysis). DT was involved as expert radiologist. The paper was mainly written by MG and BG. All co-authors provided feedback and were involved in manuscript revision.

## Conflict of Interest

The authors declare that the research was conducted in the absence of any commercial or financial relationships that could be construed as a potential conflict of interest.
